# *Orobanche aegyptiaca*-chitosan nanocomposite efficacy against the freshwater snail *Biomphalaria alexandrina*

**DOI:** 10.1038/s41598-025-02161-1

**Published:** 2025-05-20

**Authors:** Reda Ramadan Abdel-Khalek, Fathy Abdel-Ghaffar, Salwa Abdel-Hamid Hamdi, Amina M. Ibrahim, Mona Fathi Fol, Nesma Abbas Mostafa

**Affiliations:** 1 Zoology Department, Faculty of Science, Cairo University, Giza, Egypt; 2 Medical Malacology Department, Theodor Bilharz Research Institute, Giza, Egypt

**Keywords:** Chitosan, *Orobanche aegyptiaca*, *Biomphalaria Alexandrina*, Oxidative stress, Histopathological, Comet assay, Biochemistry, Biological techniques, Biotechnology, Zoology

## Abstract

As one of the neglected tropical diseases, schistosomiasis is responsible for various social and economic issues in numerous developing countries. The intermediate host of *Schistosoma mansoni* is the *Biomphalaria alexandrina* snails. A promising approach to mitigate the transmission of this disease is to use medicinal plants loaded with nanomaterials to control these snails. This research aimed to investigate the molluscicidal activity of *Orobanche aegyptiaca-* chitosan nanocomposite on *B. alexandrina* snails. The phytochemical screening of the tested plant verified its abundance of saponins, tannins, and flavonoids, which may be responsible for its cytotoxic effects. Chitosan nanoparticles were produced using the ionotropic gelation technique, while the *O. aegyptiaca*-chitosan nanocomposite was synthesized using the sonochemical approach. The characterization of the nanocomposite was carried out using TEM, XRD, and Zeta potential. The results demonstrated that the survival, fecundity (eggs/snail/week), and reproductive rates of *B. alexandrina* snails were substantially reduced upon exposure to the sub-lethal concentrations LC10 or LC25 of *O. aegyptiaca*-chitosan nanocomposite. Biochemically, it adversely affected some parameters, where it considerably (*P* < 0.05) raised ALT, AST, and ALP levels, while urea, uric acid, and cholesterol were significantly decreased compared to control snails. Furthermore, the antioxidant markers CAT, SOD, and GSH exhibited a substantial (*P* < 0.05) reduction, while MDA and NO levels increased at both sublethal concentrations. Histopathological examinations of the digestive gland of the treated *B. alexandrina* revealed a decrease in the size of the tubules, and the digestive and secretory cells degenerated. The hermaphrodite glands exhibited substantial damage in the reproductive tubules, with extensive damage to the gonadal cells, resulting in the loss of ova and sperm shapes. Also, the comet analysis showed a genotoxic effect of the nanocomposite, evidenced by significant changes in crucial comet assay parameters: tail length (TL), tail DNA percentage (TD), tail moment (TM), and olive tail moment (OTM). Conclusively, these findings confirmed the potential activity of *O. aegyptiaca-* chitosan nanocomposite as a molluscicidal agent against *B. alexandrina* to decrease schistosomiasis transmission.

## Introduction

Schistosomiasis ranks second most frequently overlooked parasitic infection after malaria^1^. *B. alexandrina* snails serve as the intermediate host for *S. mansoni*, which is extensively spread over various regions of Africa, the Middle East, and South America^2^, resulting in considerable mortality and morbidity on a global scale^3^. To remove this disease, it is necessary to disrupt the snail’s life cycle^4^. This can be achieved through chemical control or natural molluscicides^5,6^. Although chemical controlling of the intermediate host of schistosomiasis effectively decreases transmission^7^, their significant cost and potential risks to non-target organisms have prompted the utilization of natural molluscicides^8^. Furthermore, chemical molluscicide applications are expensive and require substantial financial resources, and they must be repeated annually to prevent snails from returning to water systems^9^. Recently, Egypt has implemented plant molluscicides as part of its schistosome, which requires substantial financial resources and a management program^10^. Medicinal plants have emerged as the primary source of molluscicide agents due to their cost-effectiveness and few adverse effects compared to chemical medications^11^. The plants possess several bioactive constituents exerting antibacterial, fungicidal, herbicidal, molluscicidal, and pesticidal effects^12,13^. Plants rich in saponins, flavonoids, and terpenoids exhibit significant potential for managing schistosomiasis^13^. The Egyptian broomrape or *Orobanche* is a chlorophyll-deficient, obligate root-holoparasitic plant that parasitizes vegetable and field crops associated with Solanaceae, Leguminaceae, and Brassicaceae^14^. A broomrape infestation causes oxidative stress and leads to the generation of reactive oxygen species (ROS) which can damage proteins, carbohydrates, DNA and RNA, lipids, and can result in cell death^15^. Medicinal plants growing in regions with endemic schistosomiasis could serve as valuable supplementary agents, either molluscicides or chemotherapeutics, to manage this disease. Over the last few decades, nano-enhanced approaches have been extensively evaluated for controlling parasitic infections with promising outcomes, although many challenges limit their broad implementation. Numerous researchers attempted to exploit the distinctive characteristics of nano-based delivery systems to enhance treatment^16^, develop new prophylactics^17^, and reduce the prevalence of parasitic diseases in snail hosts, resulting in significant morbidity in poor and developing nations worldwide^4^. The reduction in particle size can change materials’ physical, chemical, and structural properties, which might give rise to novel and unexpected biological effects^18^. Chitosan is an amino polysaccharide produced by partially deacetylating chitin, primarily derived from crustaceans or fungal species^19^. Its beneficial characteristics, such as low molecular weight, high water solubility, and diverse biological activities, including antibacterial, anti-inflammatory, antioxidant, and anti-diabetic properties, have received more attention^20^. Therefore, the current study aims to investigate the molluscicidal activity of *O. aegyptiaca*- chitosan nanocomposite on *B. alexandrina* snails via assessing the biological, biochemical, histopathological changes, and genotoxic effect of snail tissues.

## Materials and methods

### Ethics approval and consent to participate

The current study followed guidelines approved by the Cairo University Institutional Animal Care and Use Committee (CU-IACUC).

### Experimental snails

*Biomphalaria alexandrina* snails ranging from (9–11 mm) were acquired from the Medical Malacology Laboratory at the Theodor Bilharz Research Institute (TBRI) in Giza, Egypt. The snails were subsequently cultivated in a plastic aquarium measuring 15 × 24 × 10 cm at a temperature of 25 ± 2 °C and a light-dark cycle of 12 h: 12 h for 4 weeks before the beginning of the experiments. Each aquarium contained dechlorinated tap water (50 snails/ aquarium) and was provided with blue-green algae (*Nostoc muscorum*) and oven-dry lettuce leaves for feeding. The aquaria water was changed once a week on^21^.

### Plant materials

The Egyptian broomrape *Orobanche aegyptiaca* Pers. leaves were collected from the Giza Governorate, Egypt. They were identified by Dr. Hasnaa Hosney, a Professor of Botany, Faculty of Science, Cairo University. Using an electronic grinder, the leaves were dried and finely powdered. Voucher specimens of the tested plant were deposited at the Herbarium of the Faculty of Science, Cairo University (deposition number: Cai. 97. 465. 1373. 38). A stock solution of 5% (5 g of leaf powder in 100 ml of distilled water) was soaked at room temperature for 24 h; then it was filtered and kept till being used.

### Phytochemical tests

To ascertain the presence or absence of different phytoconstituents, such as alkaloids in Mayer’s and Draggendorff’s tests^22^, flavonoids (Shinoda test, aluminium chloride, and potassium hydroxide tests), steroids and terpenoids (Salkowski and Liebermann-Burchard’s tests), and tannins (ferric chloride and gelatin tests) according to Harborne^23^, qualitative phytochemical screening tests were conducted. Saponins were estimated to use foaming and haemolytic tests^24^, anthraquinones by Borntrager’s test, carbohydrates via Molisch’s and Barfoed’s tests, and coumarins with the Sodium hydroxide test, as per Gul et al.^25^. The outcomes were assessed through visual observation for alterations in colour or precipitation.

### Synthesis of chitosan nanoparticles

The ionotropic gelation method, as described by Calvo et al.^26^, was employed to synthesize chitosan nanoparticles. This method involves electrostatic interaction between the amine group of chitosan and tripolyphosphate. Nevertheless, 3 g of chitosan, which had been dissolved in diluted glacial acetic acid (4 ml glacial acetic acid to 296 ml doubled deionized water), was added to 2 g of sodium tripolyphosphate in 200 ml doubled deionized water. The mixture was agitated for 4 h and placed at 40 ºC for 24 h until a white gel formed. Finally, the sample was desiccated in an oven at 40 ºC for 24 h.

### Preparation of *Orobanche aegyptiaca*-chitosan nanocomposite

The *O. aegyptiaca*-chitosan nanocomposite was synthesized via the sonochemical approach, where the production and collapse of microbubbles induced by ultrasonic effects generated extreme conditions that facilitated the incorporation of *O. aegyptiaca* nanoparticles into the chitosan matrix. First, 0.1 g of *O. aegyptiaca* powder was incorporated into 100 ml of double-distilled water and agitated at 1200 rpm at 45 ºC using a magnetic stirrer for 30 min. Subsequently, 0.1 g of chitosan nanoparticles were incorporated, and stirring was prolonged for 30 min. Then, the mixture was placed in an ultrasonic device for nanocomposite synthesis under the specified conditions: Pulsate every 2 s at 85% amplitude power for 6 h.

### Characterization *Orobanche aegyptiaca*-chitosan nanocomposite

#### Transmission electron microscopy (TEM)

The morphology and particle size of the *O. aegyptiaca*-chitosan nanocomposite were analyzed using TEM (EM-2100 High-Resolution-Japan) at a magnification of 20X and an accelerating voltage of 200 kV to evaluate the two-dimensional shape and size. A droplet from a dilute sample solution was placed on an amorphous carbon-coated copper grid and allowed to dry at ambient temperature. The resultant monolayer of particles was examined with the Image J software, and many TEM pictures were captured^27^.

#### (XRD) X-ray diffraction analysis

The XRD pattern of the *O. aegyptiaca*- chitosan nanocomposite was examined using a Bruker D8 Discover XRD analyzer. The light source utilized was Cu Kα radiation, operating at a current of 35 mA and a voltage of 40 kV. The 2θ angles ranged from 5° to 80°, with a scanning rate of 0.3° perz minute. The mean crystal size was determined using the Scherrer formula:

$${\text{D}}\,=\,0.{\text{89}}\uplambda /\beta {\text{cos}}\upphi$$

where D represents the mean crystal size, λ is the x-ray wavelength, 0.89 is the shape factor, β indicates the line broadening at half maximum intensity (FWHM) in radians, and θ is the Bragg angle.

#### Zeta potential analysis

The zeta potential of *O. aegyptiaca-* chitosan nanocomposite was determined using a zeta potential analyzer (Zetasizer Nano ZS Malvern).

#### UV spectrum

UV–vis absorption spectrum was obtained using a spectrophotometer (Thermo Scientific, Helios gamma) at a wavelength range of 200–900 nm.

### Toxicity assessment of *O. aegyptiaca*-chitosan nanocomposite

To investigate the lethality of *O. aegyptiaca-*chitosan nanocomposite against adult *B. alexandrina* snails, 1000 mg/L a stock solution was prepared. Serial concentrations (70, 60, 50, 40,30, 20, and 10 mg/l) in 100 ml distilled water were set in transparent glass beakers. Three replicates of ten adult snails were exposed to 24 h for each concentration at 25 ± 2 °C and pH 7.4. Control snails were sustained under the same testing conditions in dechlorinated water. Subsequently, they were extracted and meticulously rinsed with dechlorinated tap water before being placed in containers with freshly dechlorinated tap water for 24 h of recuperation^28^. The observed mortality percentages of snails were documented, and the sub-lethal concentrations were established by probit analysis^29^. The statistical application SPSS was utilized to estimate the LC_0_ at one-tenth of the LC_50_ value^30^.

### Effect of *O. aegyptiaca-*chitosan nanocomposite on the survival rate, and egg laying capacity, reproductive rate of *B. alexandrina* snails

Snails (9–11 mm) were subjected to (LC_10_ 32.3 mg/l and LC_25_ 37.7 mg/l) concentrations of the *O. aegyptiaca-* chitosan nanocomposite for 24 h/ week, followed by 7 days of recovery in dechlorinated water for four weeks. Three replicates of 10 snails/L were saved for each concentration, with an additional set of snails serving as a control in clean, dechlorinated tap water. The water was changed twice every week. Dead snails were collected daily, and the survival rate was estimated according to Frank^31^ using the following equation:

$$\:Survival\:rate=\frac{\text{N}\text{u}\text{m}\text{b}\text{e}\text{r}\:\text{o}\text{f}\:\text{s}\text{u}\text{r}\text{v}\text{i}\text{v}\text{e}\text{d}\:\text{s}\text{n}\text{a}\text{i}\text{l}\text{s}\:}{\:Total\:number\:of\:exposed\:snails}\:\:\times\:100$$

The egg mass was collected daily by placing small pieces of polyethylene film over the aquaria, as reported by Pellegrino et al.^32^. The eggs were then preserved in small jars until they hatched. The egg-laying capacity is denoted as (Mx), representing the weekly number of eggs per snail. The reproductive rate (R0) is calculated as the summation of LxMx over the experimental duration^33^.

### Determination of biochemical indicators

#### Tissue homogenization

Control and treated snails’ soft tissues were weighed and homogenized (10%wt/vol) in an ice-cold (0.1 M Tris HCl buffer, pH 7.4) using a glass Dounce homogenizer (Swedesboro, USA). The homogenates were centrifuged for 15 min at 3000 rpm at 4^o^C. The resulting supernatant was kept at − 80^o^C.

#### Hepatic and renal markers

The obtained supernatants were utilized to assess renal function indicators (creatinine, urea, uric acid, and albumin) and hepatic markers (alanine aminotransferase (ALT), aspartate aminotransferase (AST), alkaline phosphatase (ALP), and total protein), as well as total cholesterol (TC) and triglycerides (TG) employing Bio Diagnostic kits from Egypt.

#### Oxidative stress markers

Lipid peroxidation process was assessed through the generation of malondialdehyde (MDA)^34^. The stress-induced synthesis of nitric oxide (NO) was examined as outlined by Montgomery and Dymock^35^. Reduced glutathione (GSH) is the primary intracellular thiol utilized by cells for signaling antioxidant defense^36^. Catalase activity (CAT) serves as a defense mechanism against H2O2-induced stress^37^, whereas superoxide dismutase (SOD) was assessed according to the method of Nishikimi et al.^38^ using Bio Diagnostic kits in Egypt.

#### Histopathological studies

Soft parts of both the control and treated snails were gently extracted from the shells. The hermaphrodite and digestive glands of each snail were rigorously isolated and preserved in Bouin’s solution, subsequently embedded in paraffin wax, sectioned, and stained with hematoxylin and eosin. Sections were inspected with a Zeiss microscope (Carl Zeiss Micrography GmbH 07.745 Jena, Germany) at magnifications of 200x and 400x.

#### Comet assay

Snails (9–11 mm) were subjected to LC10 or LC25 of *O. aegyptiaca*- chitosan nanocomposite, and then DNA damage was measured by single cell gel assay according to Grazeffe et al.^39^ as shown: The tissues were weighed and homogenized in ice-cold phosphate buffer at a 1:10 (w/v) ratio using a glass homogenizer for 5 min. The homogenized samples were centrifuged at 1,700 g for 15 min at 4 °C. After centrifugation, the resulting suspension was stirred for 5 min and filtered. After that, A 100 µl aliquot of the cell suspension was combined with 600 µl of 0.8% low-melting-point agarose prepared in PBS. Subsequently, 100 µl of this agarose-cell mixture was evenly spread onto pre-coated microscope slides. The slides were immersed in a lysis buffer solution (0.045 M Tris-borate-EDTA (TBE), pH 8.4, with 2.5% sodium dodecyl sulfate (SDS) for 15 min to facilitate cell lysis. After lysis, the slides were transferred to an electrophoresis chamber containing TBE buffer (without SDS). Electrophoresis was conducted under specific conditions: slides were exposed to 2 V/cm for 2 min at a current of 100 mA. Following electrophoresis, the slides were stained with ethidium bromide (20 µg/ml) and stored at 4 °C until analysis. The slides were examined using a fluorescence microscope equipped with an excitation filter of 420–490 nm and an emission filter at 510 nm. DNA migration patterns were evaluated in 100 cells, where comet tail lengths were measured from the nucleus’s center to the tail’s end at 40x. Each slide was stained with 100 µl of 1X ethidium bromide to enhance visualization. Slides were examined under an epifluorescence microscope (Zeiss, 400x magnification) with an image analysis system. Images of 50 randomly selected comets per slide were captured and analyzed for comet tail length, percentage of DNA in the tail, and tail moment, using Comet Assay V.20 software to quantify DNA damage parameters accurately.

### Statistical analysis

Statistical Processor Systems Support (SPSS) software, version 20, was employed to conduct a One-Way ANOVA on the data, followed by a Duncan post-hoc test to compare the means of the groups. The data were presented as the mean ± standard error of the mean (SEM). Statistical significance was determined at *P* < 0.05.

## Results

### Phytochemical screening analysis of *O. aegyptiaca* leaves aqueous extract

The aqueous extract of *O. aegyptiaca* leaves is rich in various phytochemical components such as alkaloids, flavonoids, saponin, tannins, carbohydrates, phenols, steroids, and terpenoids (Table 1). Also, the total phenolic content and the antioxidant capacity were 320.2 ± 4.1 and 245.2 ± 3.9, respectively (Table 2).

**Table 1:** Phytochemical screening analysis of *Orobanche aegyptiaca* leaves aqueous extract.

Secondary metabolites	Phytochemical test	Aqueous extract of O. aegyptiaca
Flavonoids	i. Shinoda	+++
	ii. AlCl_3_	+++
Saponins	i. Frothing	+++
Alkaloids	i. Dragendorff’s	+++
	ii. Meyer’s	++
Tannins	i. FeCl_3_	++
Terpenoids & Steroids	i. Salkowski	++
	ii. Libarman-Burchard’s	++
Anthraquinones	i. Borntrager’s	++
Carbohydrates	i. Molisch’s	++
	ii. Barfoed’s	++
Coumarins	i. NaOH	-

-: not detected, +: low concentration, ++: moderate, +++: high concentration.

**Table 2:** Total phenolic content (TPC) and total antioxidant capacity (TAC) of *O. aegyptiaca* leaves aqueous extract.

Parameter	*O. aegyptiaca* leaves aqueous extract
TPC (mg gallic acid equivalent/ g extract)	320.2 ± 4.1
TAC (mg AAE/g dry sample)	245.2 ± 3.9

Data was expressed as (means ± S.E.)

TPC (total phenolic content) values are expressed as mg gallic acid equivalent/g extract (mg GAE/g ext.).

AAE: Ascorbic acid equivalent.

Total antioxidant capacity (TAC) values are expressed as mg AAE/g dry sample.

### Characterization of *O. aegyptiaca-*chitosan nanocomposite

**Fig. 1:**
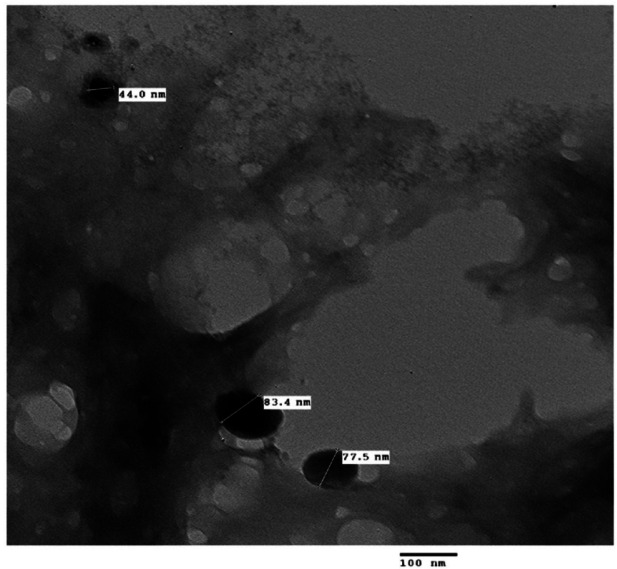
TEM image of *O. aegyptiaca-*chitosan nanocomposite.

**Fig. 2:**
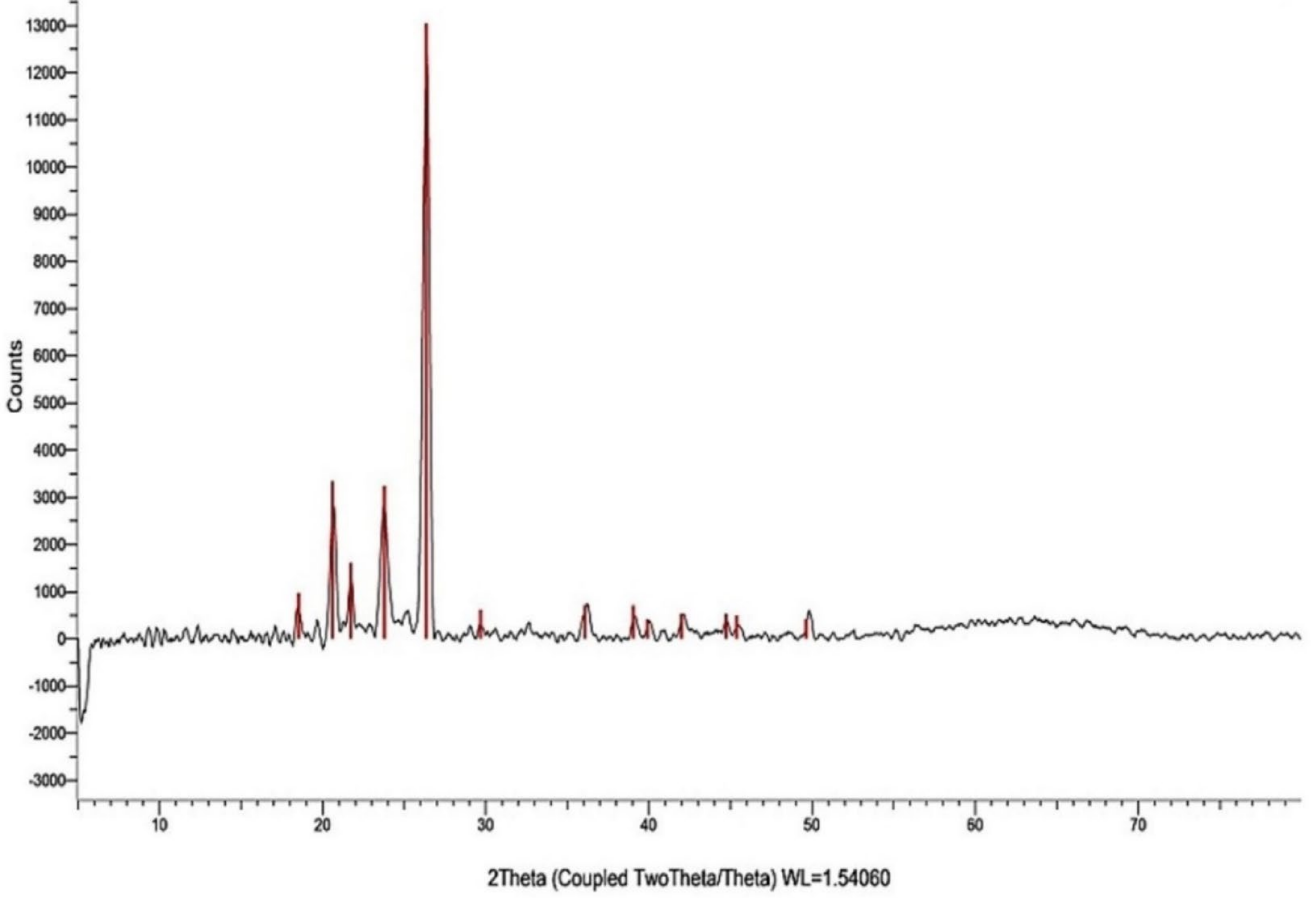
XRD pattern of *O. aegyptiaca-*chitosan nanocomposite.

**Fig. 3:**
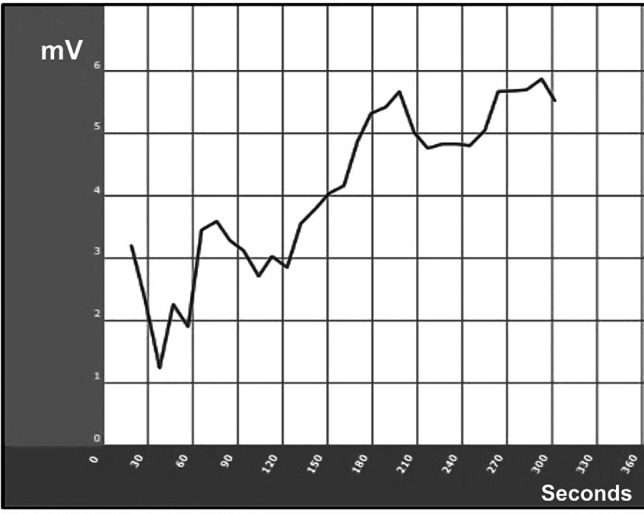
Zeta potential analysis of *O. aegyptiaca-*chitosan nanocomposite.

**Fig. 4:**
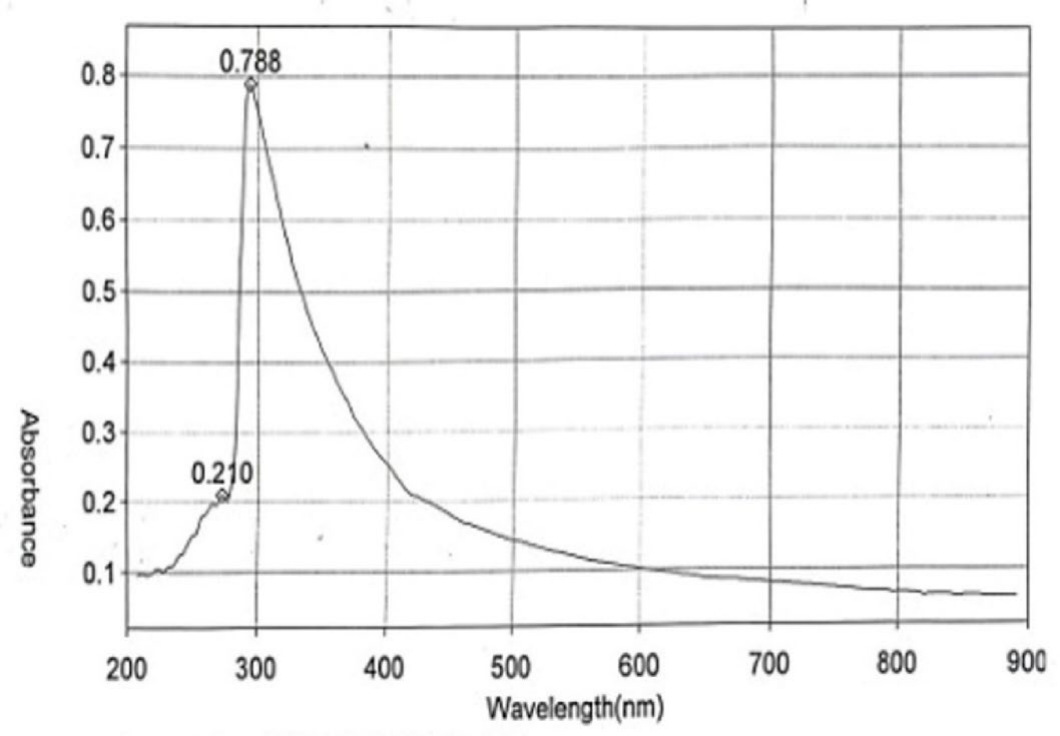
UV/Vis spectrum of concentrations of *O. aegyptiaca-*chitosan nanocomposite.

The TEM images of *O. aegyptiaca-* chitosan nanocomposite confirmed a spherical shape forming chain-like aggregates with particle size diameters ranging from 27.1 to 83.4 nm (Fig. 1). The XRD pattern illustrates the presence of five characteristic peaks at 2θ = 20.61°, 21.74°, 23.79° 26.36° and 29.69°. Other additional faint peaks appeared at 36.09°, 39.06°, 42.01°, 44.73°, 45.40° and 49.65° are probably residual chitin peaks because of the incomplete deacetylation (Fig. 2). Also, the zeta potential analysis shown in (Fig. 3) revealed a positive value of + 5.51 mV, indicating the minimal stability of *O. aegyptiaca-* chitosan nanocomposite^40^. The UV–vis spectrum of chitosan nanoparticles is shown in Fig. 4. Broad absorption bands observed at 273 and 294 nm could be related to the CO group^41^.

### Toxicity findings of *O. aegyptiaca*-chitosan nanocomposite on *Biomphalaria alexandrina*

**Fig. 5:**
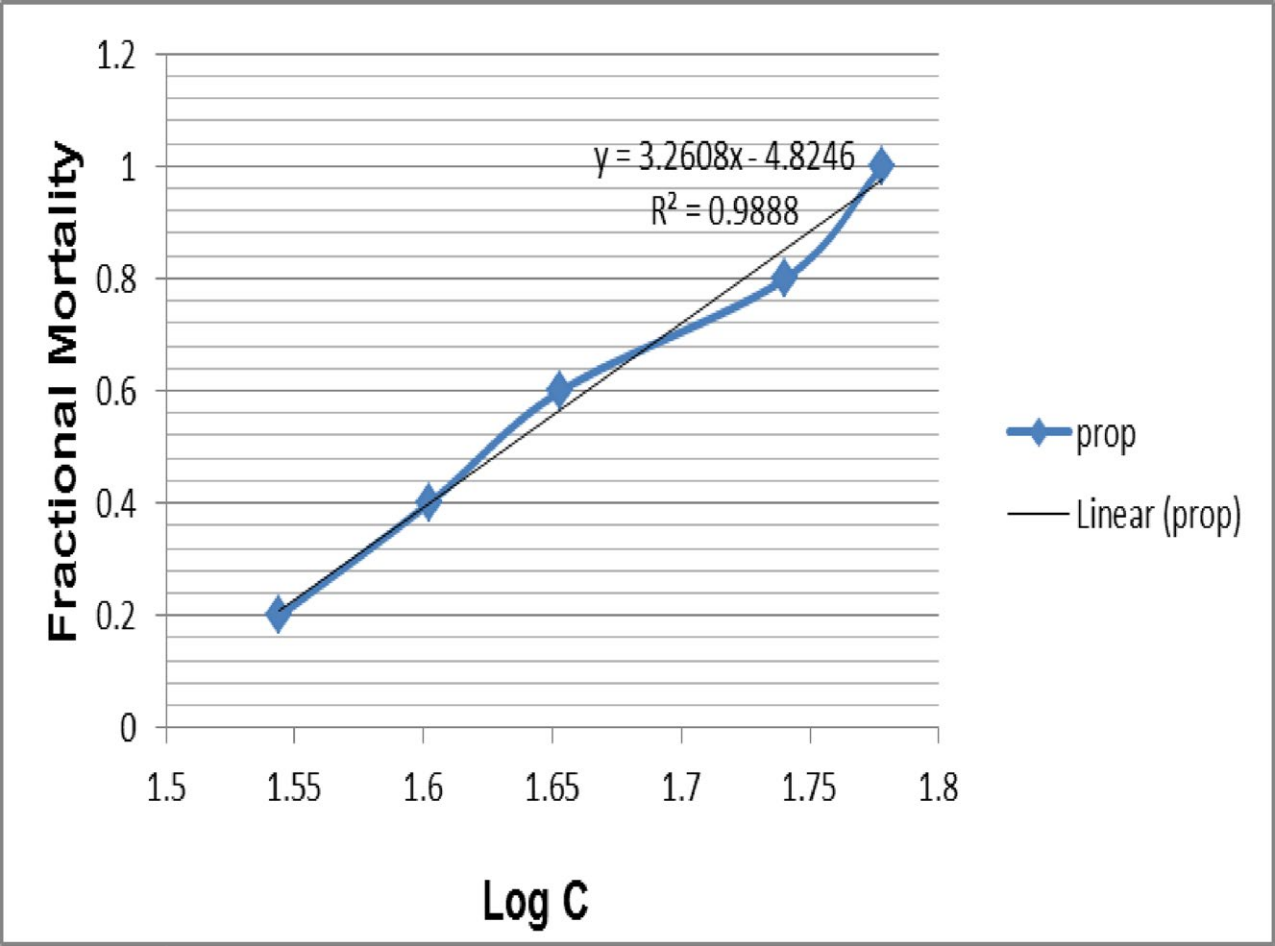
Mortality rate (%) of adult *Biomphalaria alexandrina* after 24 h exposure to various concentrations of *O. aegyptiaca-*chitosan nanocomposite against adult *Biomphalaria alexandrina* by probit analysis.

The molluscicide properties of the nanocomposite against adult *B. alexandrina* snails were determined after 24 h of exposure, followed by 24 h of recovery. As shown in Fig. 5, The obtained results revealed that the value of the median lethal concentration (LC_50_) that *O. aegyptiaca*- chitosan nanocomposite is highly toxic against *B. alexandrina* at the concentration (43.7 mg/l) and other lethal concentrations were LC_0_ (4.3 mg/l), LC_10_ (32.3 mg/l), LC_25_ (37. 7 mg/l), and LC_90_ (55.1 mg/l).

### Effect of *O. aegyptiaca-*chitosan nanocomposite on survival rate (L_x_), fecundity (M_x_), and reproductive rate of adult *B. alexandrina* snails

**Fig. 6:**
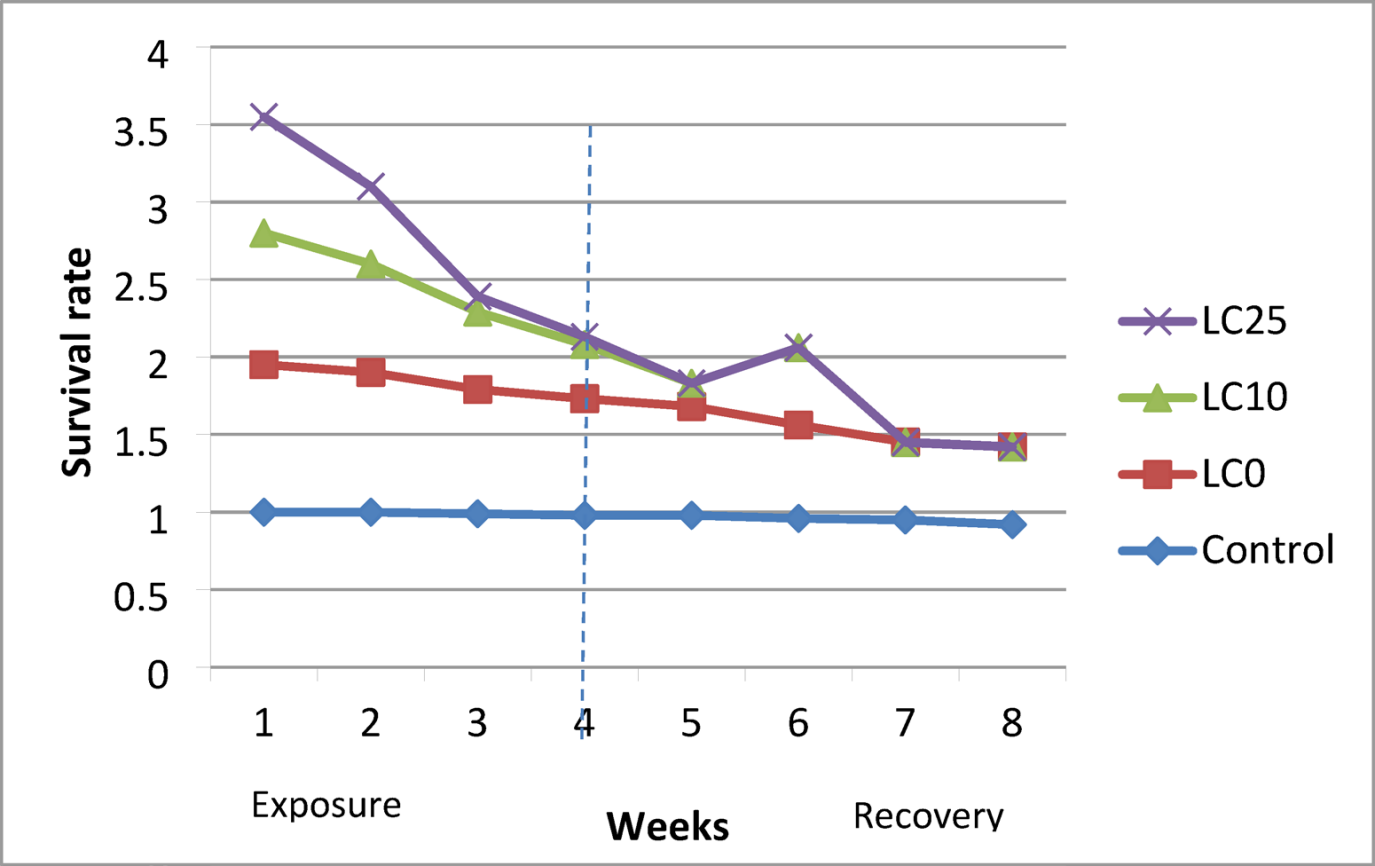
Histogram shows the survival rate (Lx) of adult *B. alexandrina* snails exposed to sub-lethal concentrations of *O. aegyptiaca-* chitosan nanocomposite for 24 h/ week for 4 weeks followed by 4 weeks of recovery.

The findings revealed that the survival rate (Lx) of snails exposed to LC0 (4.3 mg/l) was barely reduced, reaching 0.75 by the fourth week of exposure. After four weeks of recuperation, the snails survived until the end of the experiment, with L_X_ values of 0.50 in the eighth week, compared to 0.92 in the control group. Furthermore, exposure of snails to LC10 (32.3 mg/l) significantly reduced their survival rate (Lx) to 0.35 in the fourth week of exposure, compared to 0.98 in the control group. This group perished during their seventh week of recovery. Raising the concentration to LC25 (37.7 mg/l) produced a speedy and severe death among treated snails through the first 4 weeks of the experiment because their L_X_ was 0.05, then the surviving snails could not withstand this treatment and perished by the fifth week of the experiment (Table 3; Fig. 6).

**Fig. 7:**
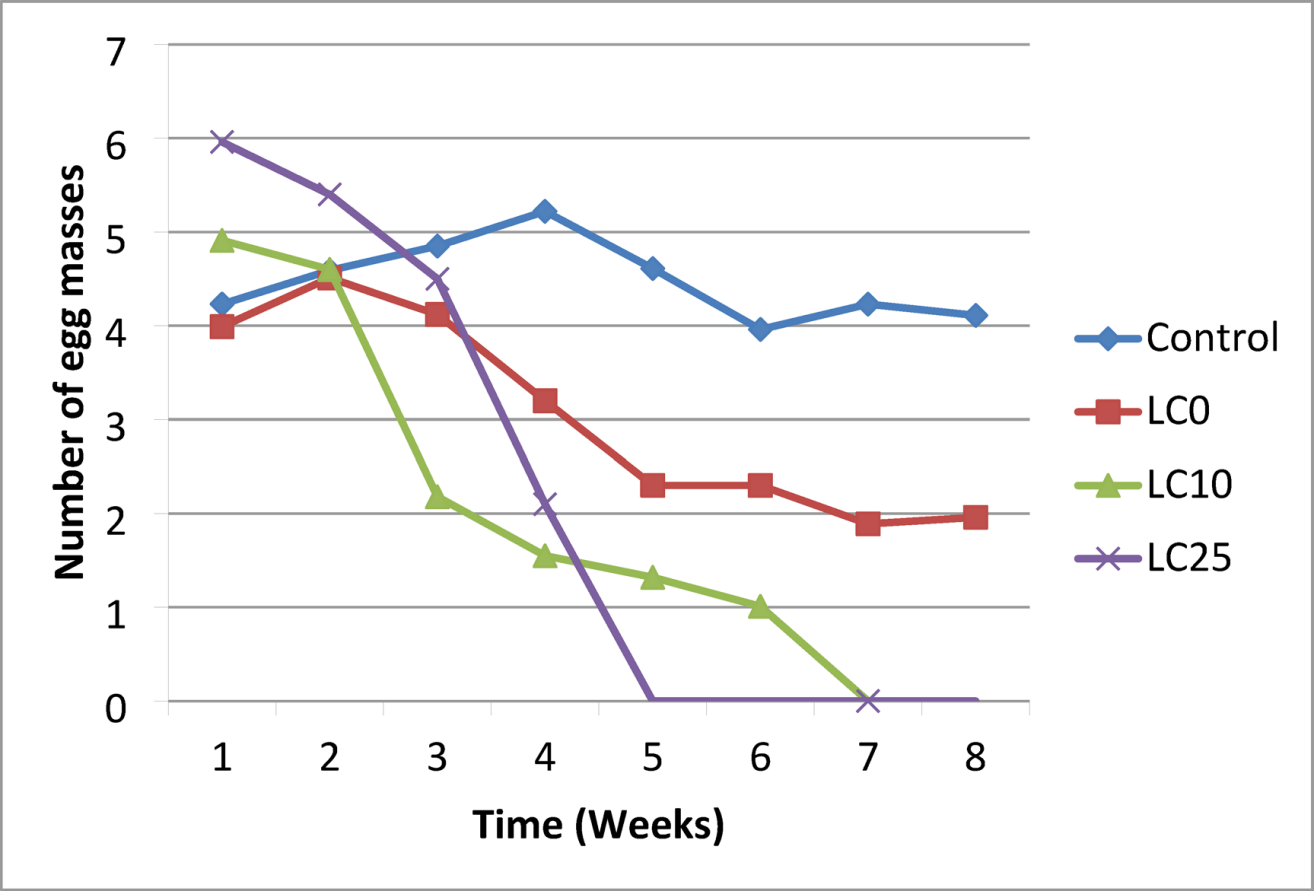
Histogram shows the fecundity (Mx) of *B. alexandrina* snails exposed to sub-lethal concentrations of *O. aegyptiaca -*chitosan nanocomposite for 24 h/ week for 4 weeks, followed by 4 weeks of recovery.

**Fig. 8:**
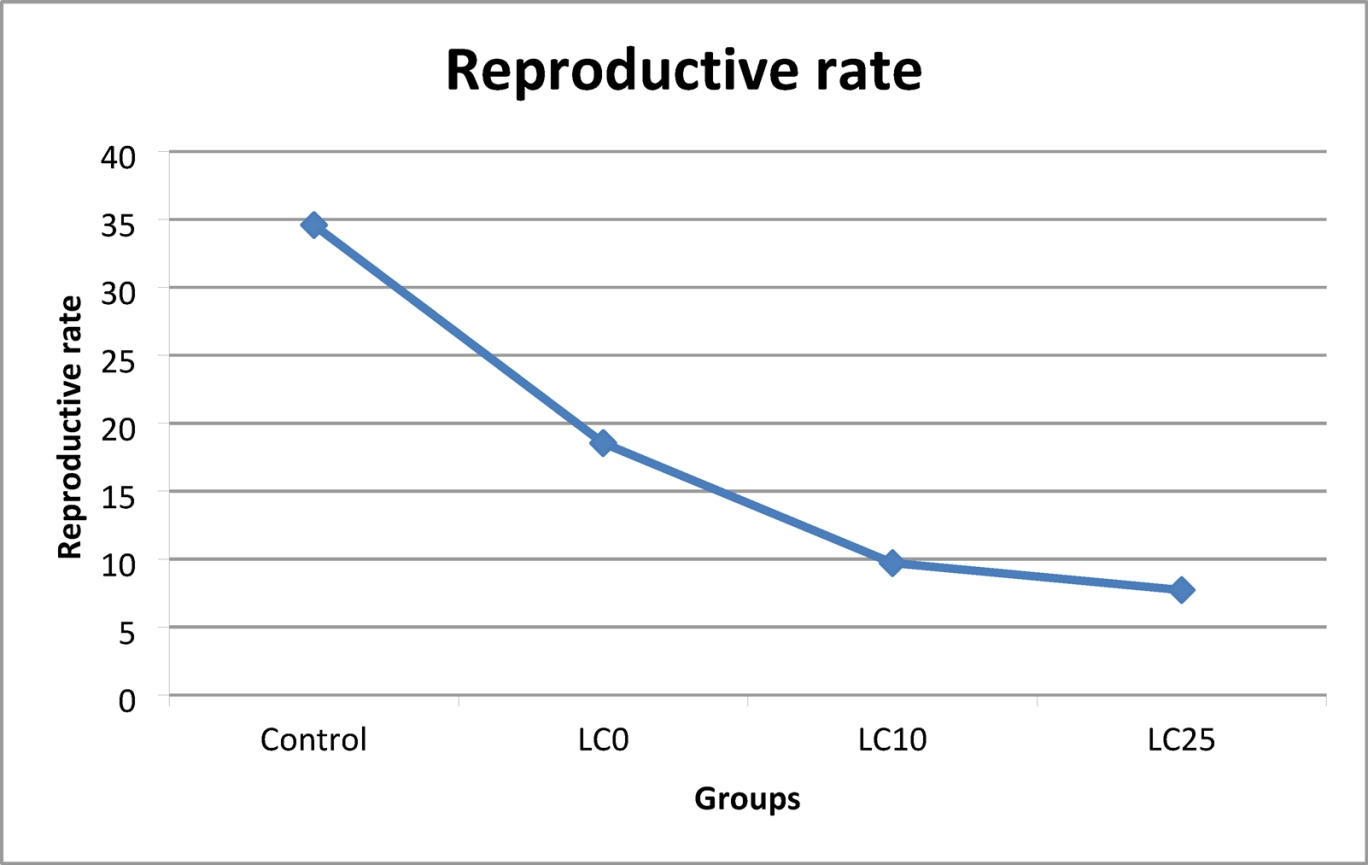
Histogram shows the reproductive rate (R_0_) of adult *B. alexandrina* snails exposed to sub-lethal concentrations of *O. aegyptiaca-*chitosan nanocomposite for 24 h/ week for 4 weeks followed by four weeks of recovery.

Additionally, snails’ fecundity (Mx) was adversely affected at the LC_0_ (4.3 mg/l) of *O. aegyptiaca-* chitosan nanocomposite, decreasing the number of laid eggs/snails/weeks throughout the experiment. During the 4th and 8th weeks, treated snails laid 3.20 and 1.96 eggs/snail/week compared to 5.22 and 4.11 eggs/control snail/week, respectively. At LC_10_ (32.3 mg/l), the snail’s fecundity was highly suppressed in the 4th week to be 1.55 eggs/snail/ week, compared to 5.22 eggs/control snail/week. Moreover, the surviving snails stopped egg-laying in the 7th week till they died in the 8th week. Increasing the concentration to LC10 (37.7 mg/l) significantly diminished the snails’ fertility in the 4th week to 2.1 eggs per snail per week, in contrast to 5.22 eggs per control snail per week. The snails ceased in oviposition in the 5th week. They perished in the 6th week (Table 3; Fig. 7). The reproductive rate (R_O_) of exposed snails revealed that it was significantly suppressed (*p* < 0.001) by snails’ exposure to the tested concentrations than the control group. At LC_0_, the reproductive rate (R_o_) was 18.54, with a percentage of 46.4% reduction in comparison with the control group (34.59). The R_o_ values of snails exposed to LC_10_ and LC_25_ were 9.71 and 7.72, respectively, compared to 34.59 for control ones, and the reduced rates under these conditions were 71.9% and 77.6%, respectively (Table 3; Fig. 8).

**Table 3:** Survival rate (Lx) and fecundity (Mx) of adult *B. alexandrina* snails exposed for 24 h/ week to sub-lethal concentrations of *O. aegyptiaca-*chitosan nanocomposite for 4 successive weeks followed by 4 weeks of recovery.

Weeks	Control			LC_0_ (4.3 mg/l)			LC_10_ (32.3 mg/l)			LC_25_ (37.7 mg/l)		
	L_x_	M_x_	L_x_M_x_	L_x_	M_x_	L_x_M_x_	L_x_	M_x_	L_x_M_x_	L_x_	M_x_	L_x_M_x_
0	1.00	3.96	3.96	1.00	3.96	3.96	1.00	3.96	3.96	1.00	3.96	3.96
1	1.00	4.23	4.23	0.95	3.99	3.79	0.85	4.91	4.17	0.75	5.96	4.47
2	1.00	4.59	4.59	0.90	4.51	4.05	0.70	4.6	3.22	0.50	5.4	2.7
3	0.99	4.85	4.54	0.80	4.12	3.29	0.50	2.18	1.09	0.10	4.59	0.45
4	0.98	5.22	5.11	0.75	3.20	2.4	0.35	1.55	0.54	0.05	2.1	0.10
5	0.98	4.61	4.51	0.70	2.3	1.61	0.15	1.32	0.19			
6	0.96	3.98	3.82	0.60	2.3	1.38	0.50	1.01	0.5			
7	0.95	4.23	4.01	0.50	1.89	0.94						
8	0.92	4.11	3.78	0.50	1.96	1.078						
R_O =_ ΣLx Mx		34.59		**18.54			***9.71			***7.72		
Reduction %				46.4			71.9			77.6		

***Highly significant from control at *p* < 0.001, compared to control.

### Biochemical parameters

Compared with the control snails, the sublethal concentrations LC_10_ (32.3 mg/l) and LC_25_ (37.78 mg/l) of *O. aegyptiaca****-*** chitosan nanocomposite showed a high significant (*P* < 0.05) increase in AST, ALT and ALP activities in snails’ tissue after exposure for 24 h as demonstrated in Table 4. However, albumin total and protein did not affect significantly (*P* < 0.05) with the sublethal concentrations LC_10_ (32.3 mg/l) and LC_25_ (37.78 mg/l) of *O. aegyptiaca****-*** chitosan nanocomposite. Also, it was observed that urea, uric acid, and cholesterol concentrations significantly (*P* < 0.05) dropped while creatinine and triglyceride appeared normal. In addition, lipid profile, cholesterol.

**Table 4:** Effect of *O. aegyptiaca-*chitosan nanocomposite on biochemical parameters.

Groups	Control	LC_10_ (32.3 mg/l)	LC_25_ (37.7 mg/l)
AST (U/ml)	10.75 ± 4.3^a^	53.3 ± 4.58^b^	46.17 ± 10.7^b^
ALT (U/ml)	1.6 ± 0.35^a^	60.22 ± 1.15^b^	72.8 ± 5.69^c^
ALP (U/L)	18.49 ± 1.0^a^	39.67 ± 0.93^b^	68.1 ± 0.99^c^
Total protein (g/dl)	1.81 ± 0.06^a^	1.74 ± 0.26^a^	1.79 ± 0.117^a^
Albumin (g/dl)	1.18 ± 0.07^a^	1.40 ± 0.089^a^	1.59 ± 0.018^a^
Urea (mg/dl)	11.86 ± 0.37^a^	6.55 ± 0.25^b^	7.28 ± 0.367^b^
Uric acid (mg/dl)	2.11 ± 0.26^a^	1.80 ± 0.88^a, b^	1.84 ± 0.19 ^a, b^
Creatinine (mg/dl)	0.33 ± 0.10^a^	0.402 ± 0.156^a^	0.736 ± 0.13^a^
Triglycerides (mg/dl)	176.5 ± 18.73^a^	177.0 ± 14.46^a^	187.1 ± 12.17^a^
Total cholesterol (mg/dl)	137.09 ± 45.7^a^	52.9 ± 6.06^b^	48.02 ± 3.63^b^

Data represented as mean ± SEM, values with different superscript letters are considered significant (*P* < 0.05).

### Oxidative stress state

The results showed a decline in antioxidant markers, including GSH content and catalase and superoxide dismutase of snail tissue after exposure to LC_10_ (32.3 mg\l) and LC_25_ (37.78 mg\l) of *O. aegyptiaca*- chitosan nanocomposite. These sub-lethal concentrations caused a substantial increase in MDA and NO levels relative to controlling snails in a concentration-dependent manner, as shown in Table 5.

**Table 5:** Effect of *O. aegyptiaca-*chitosan nanocomposite on oxidative stress markers.

Groups	Control	LC_10_ (32.3 mg/l)	LC_25_ (37.7 mg/l)
Catalase (CAT) (U/g.tissue)	38.9 ± 0.129^a^	25.09 ± 0.344^b^	14.6 ± 0.186 ^c^
Glutathione (GSH), (mg/g.tissue)	320.75 ± 7.16^a^	216.7 ± 2.17^b^	155.6 ± 9.66^c^
Superoxide dismutase (SOD), (U/g. tissue)	14315.8 ± 1288.8^a^	9672.8 ± 2990.56 ^a, b^	3980.38 ± 1506.05^c^
MDA, (µmol/g.tissue)	53.91 ± 1.18^a^	103.62 ± 0.87^b^	175.89 ± 9.16^c^
Nitric oxide (nmol/g.tissue)	13.8 ± 0.51^a^	14.6 ± 0.42^b^	18.11 ± 0.35^b^

Data represented as mean ± SEM, values with different superscript letters are considered significant (*P* < 0.05).

### Histopathological studies

#### Digestive gland

**Fig. 9:**
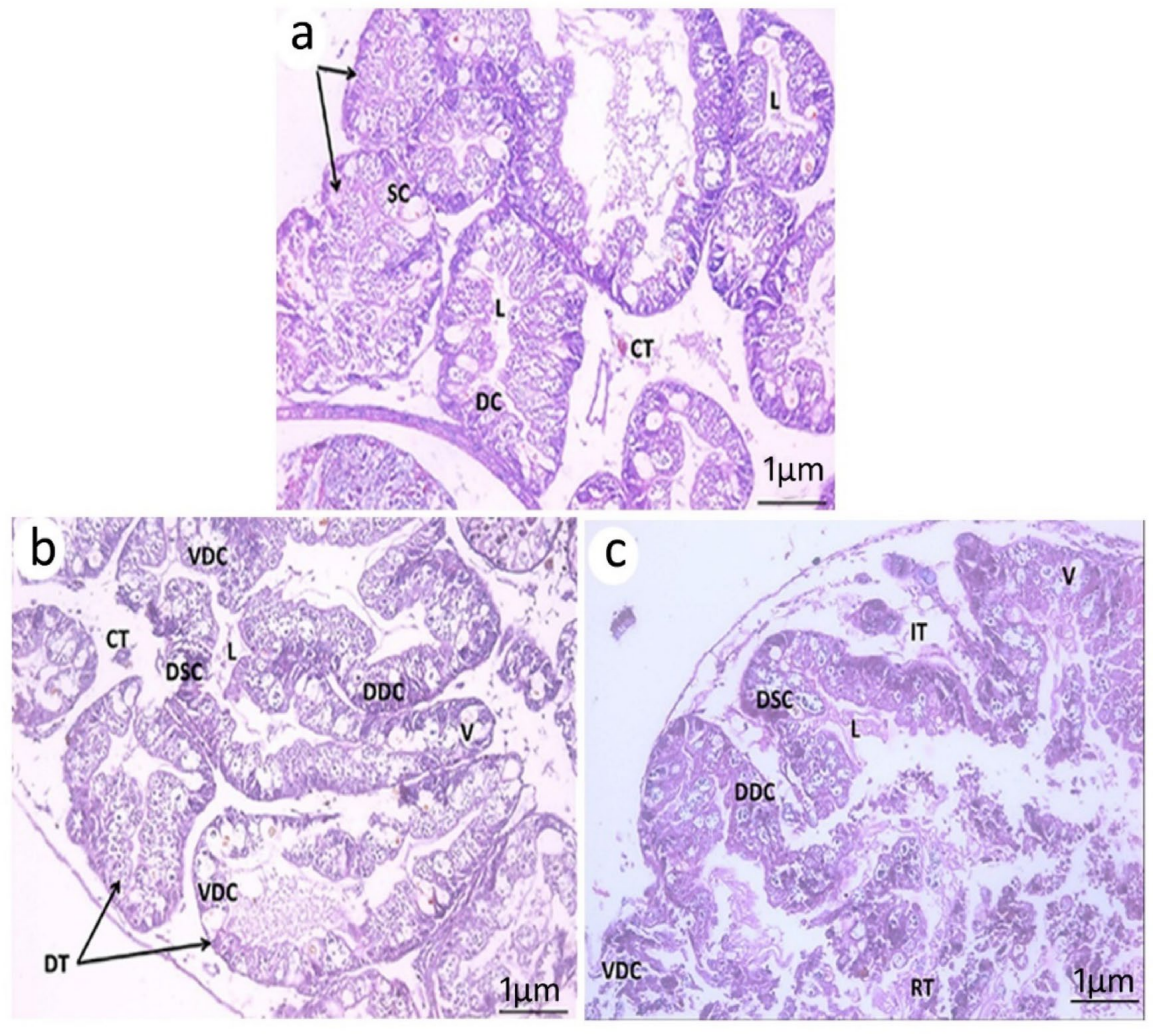
Photomicrograph of the digestive gland of *B. alexandrina*, (**a**) Control snails showed digestive tubules surrounded by connective tissue and consist of digestive and secretory cells and a lumen in the center. (**b**) Snails exposed to LC_10_ of *O. aegyptiaca*- chitosan nanocomposite showed degeneration of the connective tissue and shrinkage of some tubules with expanded lumen and some digestive and secretory cells became vacuolated. (**c**) Snails exposed to LC_25_ showed rupture of some tubules and an increase in the lumen with severe damage in the digestive and secretory cells, and epithelial cells deteriorated, resulting in the loss of cell boundaries. Connective tissue CT, Digestive cells DC, Degenerated Digestive Cell DDC, Degenerated intestinal villi DIV, Degenerated Secretory Cells DSC, Digestive tubules DT, Inter tubular space IT, Lumen L, Secretory cells SC, vacuolated V, Vacuolated Digestive Cell VDC.

The digestive gland of control *B. alexandrina* comprises two unequal lobes interconnected by loose connective tissue and consists of tubular glands of varying sizes. Each tubule is lined by columnar digestive cells with rounded apices and pyramid-shaped secretory cells (Fig. 9a). The inter-tubular space between the digestive tubules increased because of the deterioration of connective tissue and the shrinkage following exposure to LC_10_ (32.3 mg/l) of *O. aegyptiaca*- chitosan nanocomposite. Additionally, the lumen of each tubule was expanded, and some digestive and secretory cells degenerated and became vacuolated (Fig. 9b). The most devastating effect was seen at LC_25_, which included extensive degradation of the digestive tubules with expanded lumen. The secretory and digestive cells are severely damaged in most of the tubules. Additionally, epithelial cells deteriorated, resulting in the loss of cell boundaries. Digestive cells demonstrated apoptosis (Fig. 9c).

#### Hermaphrodite gland

**Fig. 10:**
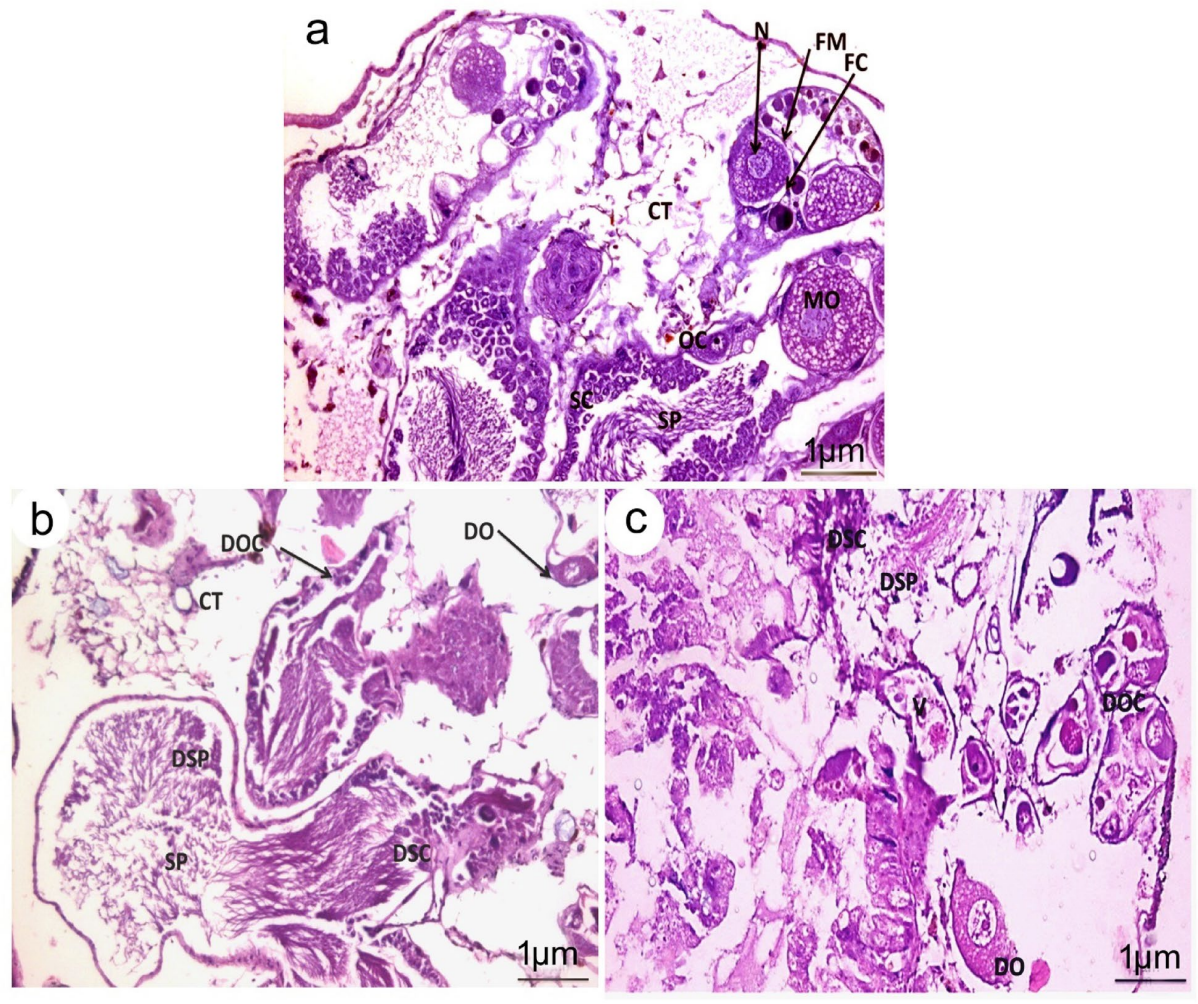
Photomicrograph of the hermaphrodite gland of *B. alexandrina* snails. (**a**) Normal snails displaying cup-shaped acini with primary and secondary oocytes, mature ovum bounded by a follicular membrane with a large nucleus appeared, the male reproductive cells were distinguished into clusters of primary and secondary spermatocytes, and fully developed spermatozoa in the lumen. (**b**) Snails exposed to LC_10_ of *O. aegyptiaca-* chitosan nanocomposite revealed marked shrinkage of the reproductive tubules with degenerations in oocytes, ova, and spermatocytes. (**c**) At LC_25_ of *O. aegyptiaca-* chitosan nanocomposite, severe damage was represented by shrinkage of the acini and compacted tubules, female gonadal cells deteriorated, the lumen of the male acini concentrated with degenerated spermatozoa. Acini A, Degenerated ovum DO, Degenerated Oocyte DOC, Degenerated spermatocytes DSPC, Follicular cavity FC, Follicular membrane FM, Mature ovum O, nucleus N, Oocytes OC, Spermatids SP, Spermatocytes SPC, Vacuole V.

**T**he hermaphrodite gland of control *B. alexandrina* snails consists of cuboidal acini linked by connective tissue. The male gametes were categorized into primary and secondary spermatocytes, with fully matured spermatozoa identified in the lumen. The female oogenic cells populated the acinar lumen with primary and secondary oocytes and mature ova encased in a follicular membrane (Fig. 10a). Sections of snails’ hermaphrodite gland subjected to LC_10_ of *O. aegyptiaca-* chitosan nanocomposite showed marked damage in some of the reproductive tubules with shrinkage, destruction, and degenerations of both male and female gonadal cells; ova and sperms with the absence of the developmental stages of spermatocytes and oocytes (Fig. 10b). At the higher concentration, LC_25,_ the gonadal cells were severely damaged, where ova and sperms vanished their shapes and distingrated. The epithelial cells of the tubules deteriorated, resulting in cell boundary loss and nuclear pyknosis (Fig. 10c).

#### Comet analysis

**Fig. 11:**
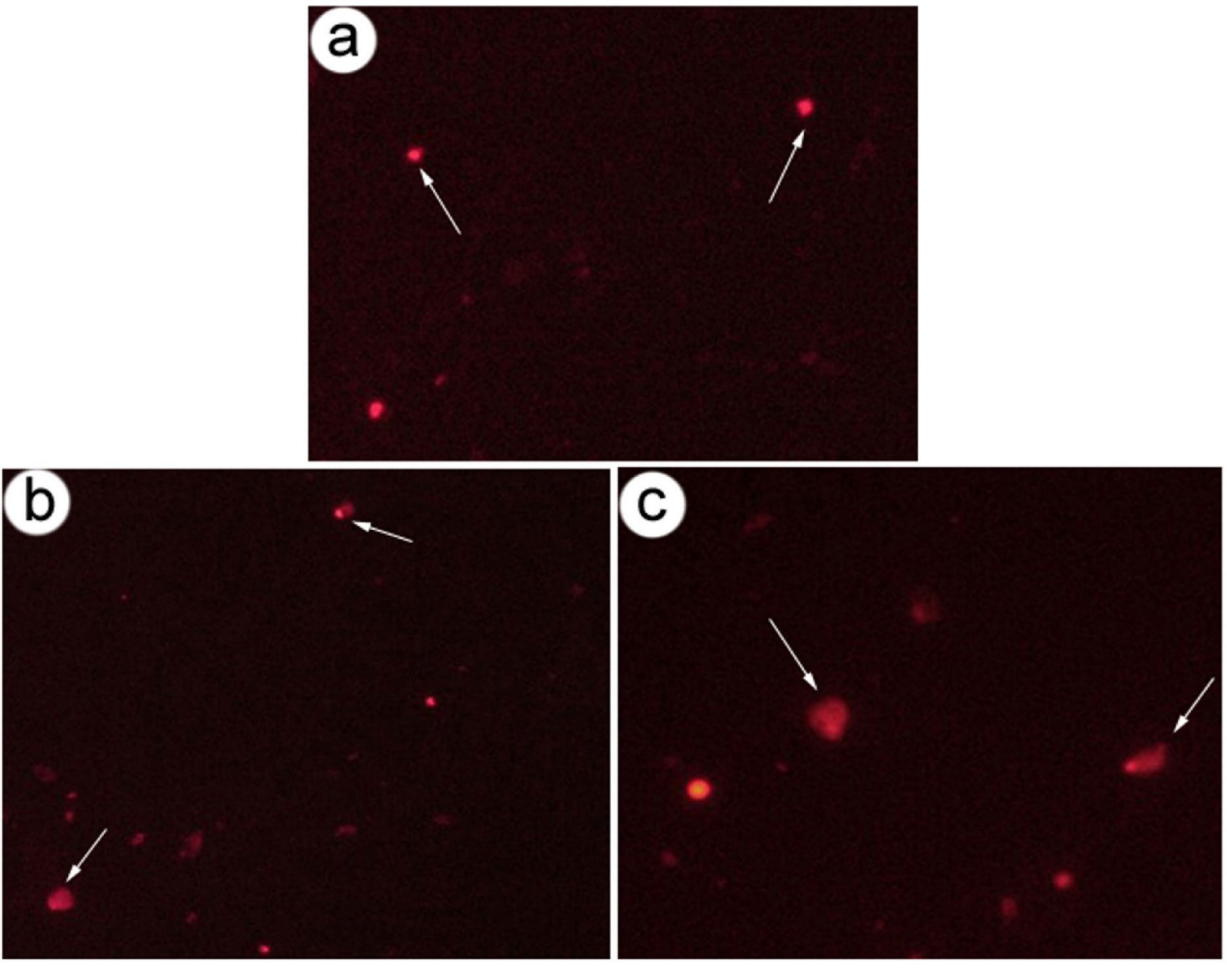
DNA damage in *B. alexandrina* snails exposed to *O.aegyptiaca*-chitosan nanocomposite, visualized by comet assay. (**A**) The control group showed minimal DNA migration, with largely intact nuclei and short comet tails (indicated by arrows). (**B**) Snails exposed to LC_10_ (32.3 mg/L) of *O. aegyptiaca*- chitosan nanocomposite showed increased DNA migration and longer comet tails, indicative of moderate DNA damage (arrows). (**C**) Snails exposed to LC_25_ (37.78 mg/L) of *O. aegyptiaca*- chitosan nanocomposite exhibited extensive DNA migration with pronounced comet tails, suggesting substantial DNA fragmentation (arrows). The comet tails represent DNA strand breaks, with tail length correlating to the degree of DNA damage.

The results of the comet assay summarized in Table 6; Figure 11 revealed significant genotoxic effects of the sublethal concentrations LC10 and LC25 of *O. aegyptiaca*- chitosan nanocomposite, with specific metrics such as tail length (TL), tail DNA percentage (TD), tail moment (TM), and olive tail moment (OTM)—highlighting the extent of DNA fragmentation and cellular impairment.

Tail Length (TL), indicative of cellular malformation and DNA strand breaks, demonstrated a substantial increase in treated snails compared to controls. The control group exhibited a mean TL of 2.960 ± 0.046 px, serving as a baseline for DNA integrity. In contrast, exposure to the LC10 concentration of *O. aegyptiaca*- chitosan nanocomposite led to a significant (*P* < 0.05) increase in TL to 4.6 ± 0.046 px. This increase was further amplified at the LC25 concentration, where TL rose sharply to 6.35 ± 1.33 px, underscoring the high degree of DNA damage induced at elevated exposure levels. Tail DNA percentage (TD) represents the proportion of DNA migrating from the head of the comet. In the control group, TD was measured at 13.08 ± 0.090%, reflecting limited DNA fragmentation. However, following LC10 exposure, there was a marked increase (*P* < 0.05) to 20.27 ± 0.79%, suggesting heightened DNA fragmentation at this concentration. The LC25 exposure group displayed an even more pronounced rise in TD to 21.49 ± 0.60% (*P* < 0.05), indicating that higher concentrations of the nanocomposite substantially exacerbate DNA damage. Tail Moment (TM), a parameter that integrates tail length and the percentage of DNA in the tail, is a sensitive marker for DNA migration and the extent of damage. TM in the control group was recorded at 0.652 ± 0.005, demonstrating baseline DNA stability. However, in the LC10 group, TM showed a notable increase (*P* < 0.05) to 1.51 ± 0.06, nearly doubling the control value and reflecting significant DNA migration and cellular genotoxic stress. The LC25 group exhibited an even more substantial rise in TM to 3.45 ± 0.288 (*P* < 0.05), signifying severe genotoxicity in response to higher nanocomposite concentration. Olive Tail Moment (OTM), which indicates the extent of DNA fragmentation, exhibited a similar trend of significant increases in treated groups. The control group showed an OTM of 1.49 ± 0.033, indicating low baseline DNA fragmentation. Exposure to LC10 resulted in a statistically significant (*P* < 0.05) increase in OTM to 2.45 ± 0.092, denoting moderate DNA damage. At the highest concentration, LC25, OTM increased sharply to 3.59 ± 0.171 (*P* < 0.05), underscoring the high degree of DNA fragmentation induced by the nanocomposite treatment.

**Table 6:** DNA damage parameters of *Biomphalaria alexandrina* after exposure to *O. aegyptiaca*-chitosan nanocomposite.

Groups	Control	LC10 (32.3 mg/l)	LC25 (37.7 mg/l)
Tail length (px)	2.960 ± 0.046^a^	4.6 ± 0.046 ^a, b^	6.35 ± 1.33^b^
% DNA in tail	13.08 ± 0.090^a^	20.27 ± 0.79^b^	21.49 ± 0.60^b^
Tail moment (Unit)	0.652 ± 0.005^a^	1.51 ± 0.06^b^	3.45 ± 0.288^c^
Olive tail moment (OTM)	1.49 ± 0.033^a^	2.45 ± 0.092^a^	3.59 ± 0.171^c^

Groups with similar superscript letters indicate no significant differences, whereas different letters denote significant differences (*P* < 0.05). All values are presented as mean ± SEM.

## Discussion

The results showed that *O. aegyptiaca-* chitosan nanocomposite TEM images revealed nanomaterial size. The XRD pattern illustrates the presence of five characteristic peaks at 2θ = 20.61°, 21.74°, 23.79° 26.36° and 29.69°. Similar peaks were observed in Chitosan structures^42^. Also, the zeta potential analysis revealed a positive value of + 5.51 mV, indicating the stability of *O. aegyptiaca-* chitosan nanocomposite. In a similar line, previous studies reported that the zeta potential values for chitosan nanoparticles usually vary from + 22 to + 88 mV, and this positive value provides better activity toward negatively charged and readily adsorbed at the cell biological membranes^43^.

Several studies indicated that using natural materials, including botanical and marine extracts, as molluscicides has received significant interest owing to the rising resistance to chemical molluscicides^44^. The present results found that the aqueous extract of *O. aegyptiaca* leaves is rich in various phytochemical components such as alkaloids, flavonoids, saponin, tannins, carbohydrates, phenols, steroids, and terpenoids. These secondary metabolites may possess potent molluscicidal properties as suggested by Augusto and de Mello-Silva^45^.

The current findings showed a significant effect of *O. aegyptica*- chitosan nanocomposite on the survival rate of *B. alexandrina* compared to the control group. After exposure to the sub-lethal concentrations, LC_10_ (32.3 mg/l) and LC_25_ (37.7 mg/l), the survival rate (Lx) considerably reduced to 0.35 and 0.05, respectively, in the fourth week compared to 0.98 for the control group. The LC_10_ group died in the seventh week of the experiment, while the LC_25_ group deceased in the fifth week. The results demonstrated a marked reduction in the survival and life span of snails correlating with increased concentration and duration of exposure. This decline in the survival rate of snails may result from metabolic disorders due to the toxic effects of the chemical components of flavonoids, alkaloids, and saponins, that precipitate on cell membrane proteins after penetration^46^. Moreover, these compounds can establish hydrogen bonds with nitrogen-free and multi-hydroxyl groups, inhibiting some enzymes critical to the organism^46^. Similarly, this investigation is consistent with the findings of Ibrahim and Abdalla^47^ who observed a decline in the survival rates of adult *B. alexandrina* post-exposure to sub-lethal concentrations of the aqueous seed extract of *Moringa oleifera.*

Transaminases enzymes, AST and ALT are critical enzymes and sensitive indicators for assessing hepatocellular damage in snails under stressful circumstances. Alkaline phosphatase benefits gastropod secretory functions and protein synthesis^2^. Concerning the biochemical changes in the tissues of *B. alexandrina* subjected to sub-lethal concentrations LC_10_ or LC_25_ of *O. aegyptica*- chitosan nanocomposite, a significant (*P* < 0.05) elevation in the activities of AST, ALT, and ALP was observed in the body tissues. Our results match those of Ibrahim and Abdalla^47^ who revealed that the aqueous seed extract of *Moringa oleifera* negatively affected some biochemical aspects, where it increased the levels of transaminases (ALT and AST). Al-Sayed et al.^48^ reported a comparable outcome, indicating that ALP activity was considerably elevated in the hemolymph of *B. alexandrina* subjected to the sub-lethal amounts of the methanol extract of *Eucalyptus globulus* and *Melaleuca styphelioides*. These alterations resulted from animal trials to restore the amino acid equilibrium in various bodily tissues and degradation of snail cells^47,49^. Furthermore, Tunholi et al.^50^ indicated that the increase in AST, ALT, and ALP activities was associated with changes in phospholipid metabolism and the elevated energy requirements of the snail. This mirrors the impact inflicted by this nanocomposite on physiological state, aligning with the findings of Abdel et al.^51^ who documented significant impairment in these essential enzymes due to exposure to synthetic and natural molluscicides.

Also, the present findings indicated a reduction in uric acid and creatinine levels following exposure to the sub-lethal concentration of the nanocomposite. In agreement with Ibrahim et al.^52^ who demonstrated an increase in uric acid and creatinine activities of *B. alexandrina* snails following exposure to chitosan-capped gold nanocomposite,

The antioxidant system was established to counteract the detrimental effects of free radicals, including the oxidation of proteins, lipids, and DNA, which may result in cell mortality and tissue malfunction^53^. In this study, the antioxidant balance was assessed in snail tissue by determining the levels of GSH, CAT, SOD, NO, and MDA. The data exhibited that antioxidants levels GSH, CAT and SOD activities substantially (*P* < 0.05) decreased following exposure to the sub-lethal concentration of *O*. *aegyptica*- chitosan nanocomposite while the NO, and MDA levels were raised. These findings are consistent with those of Ibrahim et al.^52^, who observed a decline in SOD and CAT activities of exposed snails while increased MDA levels after treating *B. alexandrina* snails with sub-lethal concentrations of chitosan-capped gold nanocomposite. Also, Ibrahim et al.^54^ discovered that treating *B. truncatus* with saponin increased NO activity.

The current investigation demonstrated that the sub-lethal concentration of nanocomposite of *O*. *aegyptica-*chitosan induced histopathological alterations in the digestive gland of *B*. *alexandrina* snails, including shrinkage of the digestive tubules and the broken down of the connective tissue, causing an increase in inter-tubular space. Also, some digestive cells became vacuolated, and the secretory cells deteriorated. The observations align with those of Abdel-Wareth et al.^55^, who illustrated that the exposure of *B. alexandrina* snails to acetone extracts of *Beauveria bassiana* and *Paecilomyces lilacinus* led to vacuolation of the digestive cells and degeneration of the secretory cells. Similarly, Ibrahim et al.^10^ recorded digestive cells rupture and degeneration of the connective tissue between tubules and deformation in the secretory cells after treatment of *B. alexandrina* with the sub-lethal concentration of the methanolic extract of *Nerium oleander* and *Tecoma stans.*

The hermaphrodite gland is one of the target tissues used to test the molluscicidal potency^56^. Herein, the histological investigation of the treated hermaphrodite gland revealed shrinkage, destruction, and degeneration of both male and female gonadal cells. At the higher concentration, LC_25,_ the gonadal cells were severely damaged, and the cell boundary loss and nuclear pyknosis were observed. Mossalem et al.^57^ observed the destruction of gametogenic cells and severe injury to hermaphrodite gland tissues by subjecting *B. alexandrina* snails to the anthelmintic plant derivative (artemether). Also, Hassan et al.^58^ identified histological alterations in the hermaphrodite gland of *B. alexandrina* snails exposed to the ethanolic extract of *Euphorbia aphylla*, *Ziziphus spina*-*christi*, and *Enterolobium contortisiliquum*.

The comet assay is widely recognized as a rapid and sensitive technique for detecting DNA damage, particularly single-strand breaks, at the individual cell level^59^. As one of the most reliable genotoxic biomarkers, the comet assay requires only a minimal number of cells to yield significant insights, making it a valuable tool in studying the effects of novel compounds and nanoparticles on DNA integrity^60^. The results demonstrated that the sub-lethal concentration of *O. aegyptiaca*-chitosan nanocomposite induced significant DNA damage in *B. alexandrina* snails, as reflected in crucial comet assay parameters: tail length (TL), tail DNA percentage (TD), tail moment (TM), and olive tail moment (OTM). The highest level of genotoxicity was observed at the LC_25_ concentration, with a substantial increase in DNA fragmentation and migration across all measured parameters compared to the control group. The increase in OTM observed in nanocomposite-treated snails aligns with findings by Ibrahim and Ghoname^8^, who reported significantly elevated OTM values in *B. alexandrina* exposed to plant-derived extracts. Similarly, Sharaf El-Din et al.^61^ demonstrated that snails treated with other nanoparticle formulations showed increased DNA migration. Our results show that exposure to *O. aegyptiaca*- chitosan nanocomposite increases tail length and DNA fragmentation in *B. alexandrina*, suggesting a substantial impact on DNA integrity. The potential toxicity of chitosan nanoparticles is mainly mediated by generating reactive oxygen species which attack cellular components, leading to oxidative stress and DNA fragmentation. causing cytotoxicity.

## Conclusion

The nanocomposite of *O. aegyptica*-chitosan was proposed in the current investigation as a potential molluscicide against the intermediate host of schistosomiasis, *B*. *alexandrina* snails. This is demonstrated by a decline in survival, fecundity, and reproduction rates. In addition, the levels of biochemical parameters, including ALT, AST, and ALP were elevated, while urea, uric acid, and cholesterol were decreased. The antioxidant markers, CAT, SOD, and GSH demonstrated a significant decrease, while MDA and NO levels increased, as well as alterations in hermaphrodite and digestive glands were observed. Also, the comet analysis exhibited a genotoxic effect of the nanocomposite. Further studies are required to confirm the effectiveness of the nanocomposite, its application, and its biotoxicity on other aquatic organisms to evaluate its target specificity and potential ecological impacts in natural water bodies.

## Data Availability

Data is available upon request from the corresponding author.
